# A Review on the Antimutagenic and Anticancer Effects of Cysteamine

**DOI:** 10.1155/2023/2419444

**Published:** 2023-09-12

**Authors:** Chun-Man Lee

**Affiliations:** Frimley Health NHS Foundation Trust, Portsmouth Road, Frimley, Camberley GU16 7UJ, UK

## Abstract

Cancer is one of the leading causes of death worldwide. First-line treatments usually include surgery, radiotherapy, and/or systemic therapy. These methods can be associated with serious adverse events and can be toxic to healthy cells. Despite the new advances in cancer therapies, there is still a continuous need for safe and effective therapeutic agents. Cysteamine is an aminothiol endogenously synthetized by human cells during the degradation of coenzyme-A. It has been safely used in humans for the treatment of several pathologies including cystinosis and neurodegenerative diseases. Cysteamine has been shown to be a potent antimutagenic, anticarcinogenic, and antimelanoma in various *in vitro* and *in vivo* studies, but a review on these aspects of cysteamine's use in medicine is lacking in the current literature. The efficacy of cysteamine has been shown *in vitro* and *in vivo* for the treatment of different types of cancer, such as gastrointestinal cancer, pancreatic cancer, sarcomas, hepatocellular carcinoma, and melanoma, leading to the significant reduction of lesions and/or the increase of survival time. Although the mechanisms of action are not fully understood, possible explanations are (i) free radical scavenging, (ii) alteration of the tumor cell proliferation by affecting nucleic acid and protein synthesis or inhibition of DNA synthesis, and (iii) hormone regulation. In conclusion, regarding the high safety profile of cysteamine and the current literature data presented in this article, cysteamine might be considered as an interesting molecule for the prevention and the treatment of cancer. Further clinical studies should be performed to support these data in humans.

## 1. Introduction

Cancer is one of the leading causes of death in the world, accounting for nearly one in six deaths, i.e., for almost 10 million deaths in 2020. First-line treatments usually include surgery, radiotherapy, systemic therapy, or a combination thereof. However, those methods can be associated with serious adverse events, impacting patients' quality of life, with variable success rates. In recent times, significant advances in finding new safe and efficient cancer have been made and have led to the emergence of promising new therapies, such as stem cell therapy, targeted therapy, ablation therapy, nanoparticles, natural antioxidants, radionics, chemodynamic therapy, sonodynamic therapy, and ferroptosis-based therapy. In this review, we will show how cysteamine (CysA), an old but high-potential drug, is providing promising results when used in many of these new emerging therapies and for myriad of cancer types.

**Figure 1:**
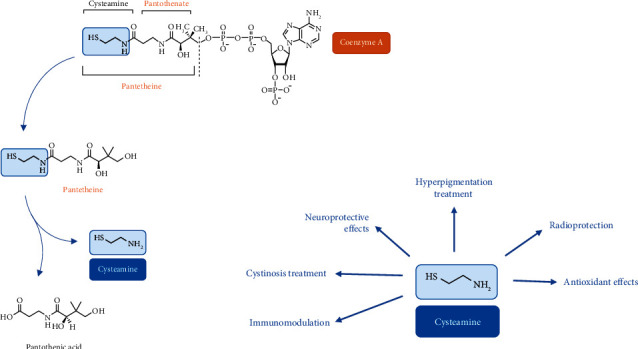
Additional effects of cysteamine other than anticarcinogenicity and antimutagenicity. Image adapted from [1, 2].

Cysteamine (CysA) is a simple, water-soluble, aminothiol (coenzyme A derivative) that is endogenously synthesized by mammalian cells. The thiol function of coenzyme A, provided by cysteamine (Figure 1), enables it to bind with the carboxyl functions of certain compounds (such as fatty acids), leading to a particularly energy-rich thioester bond that contributes directly to fat metabolism in mammals [3].

Cysteamine (CysA) has been safely used in humans for decades and has been proven to have myriads of medical uses since the 1950s (Figure 1). This substance has been used in radiation protection, growth regulation, immunomodulation, and the treatment of various conditions such as cystinosis, acetaminophen toxicity, HIV, sickle cell anemia, systemic lupus erythematous (SLE), neurodegenerative/psychiatric diseases, nonalcoholic fatty liver disease, and malaria. When used orally for the treatment of cystinosis, rare adverse effect have been reported, such as allergic rash, gastrointestinal discomfort, bone and muscular pain, hyperthermia, or lethargy, but these effects are reversible and can usually be prevented by starting the drug at a low dosage with a subsequent gradual increase over several weeks [3]. With the exception of medicines containing bicarbonate which may reduce the efficacy of cysteamine bitartrate, no drug interaction has been reported.

Small thiols such as CysA are known to be potent antioxidants since the 1980s. It was first demonstrated by Aruoma et al. that CysA was an excellent scavenger of hydroxyl radical (HO•) [4]. The antioxidant abilities of Cys to scavenge superoxide anions and hydrogen peroxide and sequestering toxic reactive aldehyde products of lipid peroxidation were suggested later [5–7]. Indeed, thiol compounds with reduced form of thiol compounds contained a functional SH group that can be oxidized to sulfenic acid (RS-O-OH), and subsequently to disulfide bonds (R-S-S-R) [8]. As thiols can react with and reduce almost all physiological oxidants before they trigger any damaging reaction, thiols serve as essential intracellular and extracellular antioxidant system [8, 9]. The antioxidant property of CysA has been used in various applications, such as (i) reduction of renal oxidation protein chronic kidney disease through reduction of the oxidative stress and of ROS generation [9] and (ii) increase of the shelf life pork meat by delaying the oxidation of heme pigment and fatty acids in muscles of pig supplemented with CysA [10].

**Figure 2:**
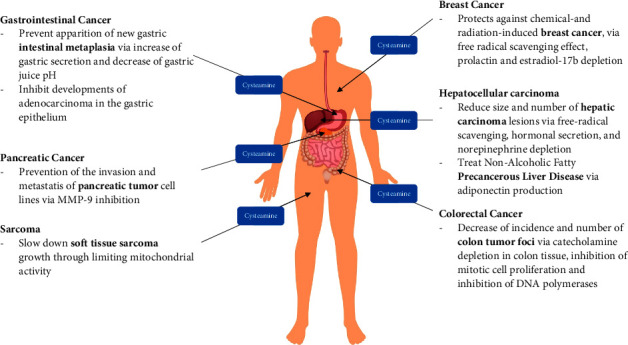
The therapeutic and preventive effects of cysteamine on various cancers (designed by Macrovector/freepik).

Cysteamine has been shown to be a potent antimutagenic, anticarcinogenic, and antimelanoma agent in various *in vitro* and *in vivo* studies [3], but a review on these aspects of cysteamine's use in medicine is lacking in the current literature. Herein, we open the study by reviewing the effect of CysA on *in vivo* cancer models after classifying tumor types based on their primary site of involvement (Figure 2). Subsequently, we discuss the radioprotective effects of CysA and its *in vitro* impact on tumoral cells. At the final part of this chapter, effectiveness of cysteamine in the form of cuprous-cysteamine nanoparticle complex (Cu-CysA NP) in enhancing chemotherapy, radiotherapy, and phototherapy targeted at cancer cells will be explored.

According to current literature, cysteamine, as a nontoxic potent antioxidant naturally present in the human body, is proved to prevent carcinogen-exposed cells from entering a cancerous state, to slow down tumor cell growth, and to reduce carcinoma lesions and tumors. It also synergizes the effect of several chemotherapeutic agents and protects against mutations induced by X- and *γ*-radiations. In addition, cuprous-cysteamine nanoparticles complex has been shown to be a potent targeted, selective and ferroptosis-base sensitizer for dynamic therapies.

Thus, cysteamine and cuprous-cysteamine nanoparticles might be considered as potentially interesting safe and effective drugs for cancer treatment and/or prevention.

## 2. Therapeutic and Preventive Effects of Cysteamine on Various Cancer Types

Cancers of the gastrointestinal system have been widely treated with CysA alone or in combination with other antitumor agents.

The correlation between ulceration and carcinogenesis in the gastrointestinal mucosa has been well documented [11–13]. Intestinal metaplasia can be induced by various stimulants, such as X-ray radiation [14] and chemical carcinogens [15].

Watanabe et al. conducted a study about the effects of gastric acid hypersecretion and hyposecretion induced by oral CysA and ranitidine, respectively, on intestinal metaplasia. This study was conducted on rats that had received X-ray radiation to induce intestinal metaplasia. Cysteamine (0.1% in drinking water) and ranitidine (0.02% of diet as a proven inhibitor of gastric acid secretion) were given to rats for two months. The incidence of intestinal metaplasia was significantly lower in the rats given X-ray + CysA compared with those treated with X-ray + ranitidine (*p*  <  0.01). In the pyloric and fundic gland mucosae of the X-ray + ranitidine group, significantly more metaplastic foci (measured with alkaline phosphatase) were observed compared with the X-ray + CysA given rats [16].

Even though the mechanism of action of the protective effect of CysA was obscure in this study, Watanabe et al. proposed that epithelial cell differentiation in the esophagus, stomach, and duodenum is dependent on the pH of gastric mucosae. In separate studies, Watanabe et al. suggested that gastric pH elevation is a major risk factor for the occurrence of intestinal metaplasia [14, 16, 17]. Meanwhile, in the specified study, X-ray radiation was hypothesized to induce parietal cell disappearance in fundic gland mucosae, thereby leading to a pH elevation in the gastric content and consequently inducing intestinal metaplasia. Since the oral consumption of CysA correlates with increased gastric acid secretion, pepsin activity, and serum gastrin levels [18–20], the antimetaplastic effect of CysA was speculated to be via the induction of a decrease in gastric juice pH.

Tatsuta et al. demonstrated that the long-term administration of CysA inhibits the development of adenocarcinomas induced by N-methyl-N '-nitro-N-nitrosoguanidine (MNNG; a chemical carcinogen) in the gastric epithelium of inbred Wistar rats. MNNG was administered to rats for 25 weeks to achieve a highly differentiated adenocarcinoma incidence of up to 80%. The application of 25 and 50 mg per kg CysA until week 52 decreased both the incidence and numbers of adenocarcinomas in the glandular epithelium of the rats' stomachs. In a control group of rats receiving MNNG + NaCl, the incidence of gastric cancer was 80%. On the other hand, in the MNNG + CysA 25 mg/kg and MNNG + CysA 50 mg/kg treatment groups, the incidence of gastric cancer was 31.6% and 33.3%, respectively [21].

Tatsuta et al. clarified the relation between CysA-induced hypergastrinemia/gastric acid hypersecretion and gastric cancer suppression, by testing the effect of a combination of propranolol + CysA vs cimetidine + CysA vs CysA alone on MNNG-administered rats [22]. Propranolol, as a beta 1-adrenergic blocker, induces gastric hypersecretion [23] and reduces the gastric mucosae thickness by reducing parietal cell numbers [24]. The researchers used the labeling index of gastric mucosae to give analytic feedback on interference with cell growth kinetics; the administration of propranolol + CysA seemed to significantly boost the suppressive effect of Cys against gastric carcinoma in comparison with isolated CysA treatment. At the end of the MNNG study, only the rats treated with propranolol + CysA or CysA alone had a significant reduction of gastric cancer; only 9% of rats in the propranolol + CysA group and 45% in the CysA group had gastric cancer versus 80% in the placebo group [22], for which two possible explanations are given: firstly, the accelerated gastric acid secretion of CysA in combination with propranolol seemed to potently deteriorate the gastric cancer foci considering the fact that gastric acid can ulcerate cancerous lesions [25, 26]; secondly, it is hypothesized that gastric acid secretion either by CysA alone or propranolol + CysA can affect gastric mucosal cell proliferation in a manner similar to that shown later in 2021 by Koh et al. on propranolol alone [27], thereby inhibiting the cancerous foci. On the other hand, cimetidine (an antihistaminic agent with high potency against H2 receptors) + CysA administration gave rise to a slight increase in the incidence of gastric cancer (57% of rats had gastric cancer at the end of the study). The administration of cimetidine + CysA caused the neutralization of the gastric acid hypersecretion but not the hypergastrinemic effect, although Tatsuta et al. concluded that hypergastrinemia alone cannot inhibit gastric carcinogenesis induced by MNNG [22].

Based on the observations of Watanabe et al. and Tatsuta et al., four possible explanations on the pathophysiology of tumor cell suppression by CysA were provided: (i) given that CysA increases both serum gastrin level and gastric acid secretion [28], tumor cell suppression might be due to the hypergastrinemic state and interference with growth kinetics since it has been established that the gastrin hormone has a trophic effect on gastric mucosal cells [29]; (ii) as shown by Badger et al., CysA consumption induces the secretion of many hormones including growth hormone, thyrotropin, B-endomorphin, prolactin, and gonadotropin [30], while Szabo and Reichlin reported that CysA induces immune-reactive somatostatin depletion in the GI tract mucosa [31]. A hormonal mechanism might therefore be involved in the induction of gastric cancer; (iii) CysA induces histamine secretion thereby providing antiadenocarcinoma effects because prolonged administration of histamine significantly reduces the incidence of cancer in the rat stomach [32]; and (iv) CysA alters nucleic acid and protein synthesis [33] via adenyl cyclase activation and increased intracellular cAMP. Ryan and Heidrick (1968) found that the application of cAMP suppresses DNA synthesis in tumor cells. Thus, CysA might affect gastric mucosal cell proliferation.

### 2.1. Colorectal Cancer

Despite the availability of multiple studies on the anticarcinogenic effects of CysA against gastric cancer via multiple suggested mechanisms of action, there is only one *in vivo* study in the literature that discusses the effect of CysA against colorectal cancer.

Tatsuta et al. showed that for colon cancer induced by azoxymethane (AOM) in Wistar rats, the subcutaneous injection of CysA significantly decreased both the incidence and number of tumor foci in the rats' colons. In the control group (AOM + NaCl), colon tumors were present in 94% of the surviving rats. In the treatment group (AOM + CysA), colon tumors were found in only 41% of the surviving rats. At the end of the study (week 40), rats that had been treated with CysA had significantly lower body weights. Furthermore, no metastatic foci were found in the peritoneum or lymph nodes of rats in either group [34].

The norepinephrine hormone, synthesized by the sympathetic nerve chain, activates crypt-cell proliferation in the large and small intestines [35, 36]. Cysteamine administration reduces norepinephrine levels in the stomach, duodenum, and brain tissues [37]. Tatsuta proposed that catecholamine depletion in colon tissue due to prolonged administration of CysA is the key mechanism of tumor cell alteration in rats [34]. Cysteamine has been shown to directly inhibit mitotic cell proliferation *in vitro* in cultured HeLa cells [38]. Considering this fact, Tatsuta's study suggests that the inhibition of DNA polymerases and cell cycle signals might be another mechanism through which CysA inhibits colon carcinogenesis [34].

### 2.2. Pancreatic Cancer

To date, pancreatic cancer has the highest mortality rate among the various cancers in developed countries. It is also considered as a highly metastatic and difficult-to-treat cancer [39].

In the study by Fujisawa et al. in 2012 that investigates the antitumor and antimetastatic properties of CysA in a human cancer cell model, it was proven that CysA suppresses both the invasion and metastasis of tumor cell lines. In this investigation, human pancreatic cancer cell lines were implanted in two immunodeficient mice; subcutaneous injections of CysA (twice daily) were commenced four days later. At the end of the survey (day 30), both primary and metastatic foci of tumors were checked by size and weight. The mice showed significantly (90%) less metastatic nodules when high-dose CysA (250 mg/kg/day) was administered; the total weight of the metastatic nodules also decreased by 90%. However, the size and weight of the primary tumors remained unchanged [40]. These findings support the use of CysA in combination with other antitumor drugs. Furthermore, CysA also decreased the rate of aggressive pancreatic cancer ascites. At follow up, CysA treated mice had longer survival time in comparison with the control group. In the same study, using 10 different pancreatic cancer cell lines *in vitro*, CysA decreased either the invasion or migration of cancerous cells at a noncytotoxic dose [40].

Cysteamine inhibits tumor migration and metastasis through the reduction of matrix metalloproteinases' (MMPs) activity both *in vivo* and *in vitro* [40]. MMPs are a group of endopeptidases in charge of cell growth and turnover kinetics, tissue remodeling, wound healing [41], and cancer cell line dynamics [42]. MMP-9 is the exclusive agent in charge of pancreatic cancer metastasis to the liver [43]. Clinically, CysA has been identified as a potent MMP inhibitor with little or no side effects. Also, in two immunodeficient mice, CysA (at 100 and 250 mg/kg/day) decreased MMPs' activity, especially that of MMP-9. Considering the fact that CysA has no proven clinical or preclinical side effects, Toshio Fujisawa et al. recommended this drug as a monotherapy before surgery to prevent metastasis or as an adjuvant in the advanced stages of cancer [40].

Similar results were obtained by Suzuki et al. in 2017 when studying the use of cysteamine as a treatment of pancreatic cancer. The use of cysteamine was shown to directly inhibit MMPs enzymatic activity both in vivo and *in vitro*, thus inhibiting cell migration and cell invasion. However, the use of cysteamine seemed to have little effect on the primary tumor's size and thus should be used in combination with other antitumor drugs. It is worth noting that the MMP inhibition of cysteamine is less efficient than other specific MMPs inhibitors but cysteamine would be better tolerated *in vivo* [44].

### 2.3. Hepatocellular Carcinoma

In the rat model of hepatocellular carcinoma induced by N-nitrosomorpholine (NNM), prolonged alternative day subcutaneous injection of CysA significantly reduced neoplastic and preneoplastic liver lesions in both number and size. Transectional data on hepatic lesions revealed that CysA treated mice had significantly less lesions (25.5 and 15.9 number/cm^2^; GGT-positive and G6PD-positive lesions, respectively) in comparison with a control group (22.3 and 31.4 number/cm^2^). Histologic analysis of lesions also revealed that CysA treated mice had significantly smaller and significantly fewer hepatic lesions. Rat survival time was not checked in this study [45].

Although further investigations are required to identify the exact mechanism behind CysA's inhibition of hepatocarcinogenesis, a number of explanations have been suggested: (i) the free-radical scavenging feature of CysA that has been shown in radiation-based studies; (ii) CysA consumption induces the secretion of many hormones including growth hormone, thyrotropin, B-endomorphin, prolactin, and gonadotropin [31, 46, 47], which may directly or indirectly be in charge of hepatocyte growth behavior and hepatocarcinogenesis; (iii) CysA has been shown to alter nucleic acid and protein synthesis [33] due to adenyl cyclase activation and increased intracellular cAMP [26]; and (iv) considering the close association between adrenergic hormones and liver regeneration after partial hepatectomy [48, 49] and given that CysA induces norepinephrine depletion in many tissues including the liver, the anticancer effect of CysA in the rat model of hepatocarcinogenesis induced by NNM may be due to decreased liver levels of norepinephrine [45].

Although the most common liver precancerous condition is considered liver cirrhosis, any chronic condition that predisposes this organ to a degeneration/regeneration state is considered as a hepatocellular carcinoma risk factor, namely, alcoholism, nonalcoholic fatty liver disease (NAFLD), and viral and autoimmune hepatitis [50]. NAFLD is the most common condition that is named as a “chronic liver disease” in North America. In this disease, the chronic accumulation of triglyceride droplets in hepatocytes leads to infiltration of inflammatory cytokines as well as the development of pericellular fibrosis [51]. Cysteamine is known to be effective in treating NAFLD as a precancerous liver condition in human models. In one study, CysA decreased both ALT and AST levels over a nine-day period of administration in patients with NAFLD [52]; it was reported that AST and ALT remained below baseline levels 24 weeks after withdrawal of the drug, suggesting that CysA inhibits fibrosis due to its reactive oxygen species scavenging effect and inhibition of transglutaminase activity [52]. Cysteamine administration also causes adiponectin production, which has an antiinflammatory effect that contributes to the treatment of NAFLD [51].

Systemic lupus erythematosus (SLE) is a systemic autoimmune disorder that commonly involves the liver; it triggers various inflammatory cascades and produces oxidative stress in this organ. In a mice model of SLE, CysA reduced both liver inflammation (decreases AST and ALT levels) and abnormality [53]. It is more or less evident that CysA can decrease the incidence of hepatic cancer; further trials featuring the application of CysA in precancerous liver states seem highly necessary.

### 2.4. Breast Cancer

It is now statistically documented that breast cancer is the most common cancer among the female population around the world. Several risk factors are of importance in the epidemiology of breast cancer, including unhealthy lifestyle, long time fertility, and obesity [54]. Radiation is an agent known to experimentally induce mammary tumors in animal models [55]. Carcinogenesis induced by radiation is mostly due to augmentation of oxygen radicals in the exposed tissue.

Cysteamine is considered a major radioprotective agent due to its feature of scavenging free radicals [56]. In one study, the consumption of CysA prior to and after the administration of 7,12-dimethylbenz[a]anthracene (DMBA; a toxic and carcinogenic agent) significantly decreased the number of mammary tumors in Sprague–Dawley rats. DMBA mediates oncogenic changes via the production of free radicals in the exposed tissue. Marquardt et al. revealed that in the course of 11 months of treatment with DMBA + CysA, the number of mammary tumor-bearing rats was significantly lower. These results indicate that CysA is a potent protective agent given its radical scavenging character [57].

In a separate study, the administration of low-dose CysA prior to whole-body irradiation (1.5 Gy) followed by exposure to diethylstilbestrol, a tumor promoter, significantly decreased the incidence of tumor cell initiation (in the course of 1-year) in the mammary glands of pregnant rats. The incidence of mammary tumor was 20.8% in the irradiated rats treated with CysA, while the control group (saline injected rats exposed to same irradiation dose of 1.5 Gy and to diethylstilbestrol) had a mammary tumor incidence of 71.4% [58].

Beside the free-radical scavenging properties of CysA, which is the key mechanism of action in chemoprevention, there are several other explanations. One possible reason for this antitumor effect is serum prolactin depletion during irradiation. The administration of CysA has been shown to decrease plasma prolactin levels rapidly [59], and the mammary glands' prolactin function has a correlation with the incidence of mammary tumor caused by radiation [60]. Also, prolactin-stimulated differentiation during lactation enhances tumor initiation by radiation [58].

Herein, another possible explanation of the protective effect of CysA is estradiol-17b serum depletion after irradiation in pregnant rats due to the fact that estrogen accelerates tumor initiation by radiation [61] or since estrogen-demanded development in pregnancy sensitizes the mammary glands to radiation induced tumor-genesis [62]. In one study, the administration of CysA or WR-2721 (amifostine, another radical scavenging aminothiol) reduced the number of both ER/PGR^+/+^ and ER/PGR^−/−^ tumors induced by radiation in pregnant rats [58].

### 2.5. Sarcoma

Sarcoma is a cancer of connective tissue (e.g., fat and muscle), accounting for about 1% of cancers in the United States [63]. The effect of CysA on sarcoma is an issue yet to be studied. According to the literature, a range of standard studies have explored the molecular attitude of endogenous CysA on tumor growth and metastasis.

Pantetheinase is a well-studied physiological system for tissue stress tolerance programs. It induces the breakdown of pantetheine into two major molecules that regulate several cellular behaviors: firstly, pantothenate (vitamin B5), which is absorbed from intestinal cells to synthesize coenzyme A, thereby regulating cell mitochondrial metabolism; secondly, CysA, a simple aminothiol precursor for the synthesis of hypotaurine and taurine, essential aminoacids to maintain brain function [7] (Figure 1). The enzymatic activity of pantetheinase has a key role in tumor cell growth. Metastatic tumors (e.g., sarcoma) favor glycolysis (a nonmitochondrial path) followed by pyruvate reduction to lactate rather than oxidative phosphorylation for growth regulation. This feature is called the Warburg effect and is the attitude that most aggressive tumors follow. Endogenous CysA production can limit glycolysis through reduction of lactate levels in tumor cells both *in vitro* and *in vivo*. Thereby, CysA antagonizes the Warburg effect during tumor cell metabolism [64] and can slow down soft tissue sarcoma growth through limiting mitochondrial activity. These data indicate that the regulation of pantetheine derivatives may turn into an interesting novel pathway for cancer treatment [65].

## 3. The Radiation Protection Effect by Cysteamine

Since the 1950s, cysteamine has been recognized for its antimutagenic properties against various types of radiation, including X-rays, *γ*-rays, and UV light.

Abundant data are available in the literature regarding the radioprotective effect of cysteamine against mutations induced by X-rays. In 1955, Devik and Lothe demonstrated the antimutagenic effect of cysteamine against chromosomal aberrations in the bone marrows of mice that were irradiated with X-rays. In that study, the researchers intraperitoneally injected albino mice with fresh preparations of 3 mg of cysteamine (in 0.2 mL of distilled water) 8 to 12 minutes prior to radiation exposure; the investigation revealed the protective effect of CysA against anaphase abnormalities and death induced by 200 R (≈1.66 Gy) and 1100 R (≈9.13 Gy) total body radiation exposure, respectively [66]. Later in 1961, Lüning et al. conducted a similar investigation; 4 mg of cysteamine (in 0.3 mL physiological saline) was intraperitoneally injected into the mice 15 min before exposing the subjects to either 300 (≈2.5) or 600 R (≈5 Gy) of X-rays. The researchers found that cysteamine provided protection for mice spermatozoa cells against lethal X-ray induced mutations [67]. Kølmark demonstrated the protective effect of cysteamine against both X-ray induced mutation and mortality in *Neurospora crassa* [68]. In this study, maximal protection was reported in cysteamine concentrations between 0.020–0.025 M and X-ray doses below 50 kR (≈415 Gy). In an *in vitro* study conducted on a human kidney cell line, 2–8 mM cysteamine displayed anti mutagenic effects against chromosomal damage induced by 200 rad (2 Gy) of X-rays [69]. In 1967, Mikaelsen and Pedersen demonstrated the significant protective effect of cysteamine *in vivo* against X-ray induced chromosomal aberrations in Allium cepa root meristem cells. These researchers made use of 100 R (≈0.83 Gy) of X-irradiation, reporting that 0.001 M cysteamine offered radioprotection during early interphase (at the G1-stage) [70]. Lohman et al. investigated in 1970 the protective effect of various concentrations of cysteamine against different X-rays both *in vitro* (human T-cells in tissue culture) and *in vivo* (*Escherichia coli*). In this study, 0–3 krad (0–30 Gy) of X-irradiation was applied to monolayers of T-cells after 32 mM cysteamine had already been added in the preceding 10–30 min, while 0–40 krad (0–400 Gy) of radiation was applied 10 minutes after incubation of *E. coli* cells with 44 mM cysteamine in modified M63 culture medium at 37°C. In both cases, cysteamine offered significant protection against DNA strand breaks induced by the specified doses of radiation [71]. In 1972, Roots and Okada also evaluated cysteamine for its *in vitro* radioprotective properties, finding that cysteamine protected the DNA of cultured mouse leukemia cells from single-strand scissions that were induced by 10 krad (100 Gy) of X-irradiation; they suggested that a free radical scavenging mechanism was behind this radioprotection [72].

Although must studies in the literature have focused on the protection offered by cysteamine against X-rays, other forms of radiation have also been investigated. Stern et al. demonstrated the antimutagenic effect of cysteamine against corays in *Escherichia coli*. In this study, the researchers used 0.1 M cysteamine and found that it provided a protection ratio of 2.4 against radiation doses in the range of 10–80 krad (100–800 Gy) compared to the control [73]. In another study, Radman et al. looked at the *in vivo* protection offered by cysteamine against UV light in irradiated Lambda phage. They found that 0.1% cysteamine provided protection against repairable UV-induced DNA lesions in bacteria, which were suggested to occur as a result of pyrimidine dimer formation [74].

On the other hand, the antimutagenicity of cysteamine against chemical mutagens has also been the subject of a number of studies. In 1973, Becher and Gebhart demonstrated cysteamine's strong, dose-dependent, *in vitro* protection of human lymphocytes from chromosomal damage induced by trenimon, an anticancer drug that acts via alkylation and is well-known for its clastogenic effects [75]. Rosin and Stich reported that cysteamine had good, dose-dependent antimutagenicity against mutations induced by 50–100 *μ*M N-acetoxy-2-acetylaminofluorene in *Salmonella typhimurium* [76]. In another study, Negishi et al. reported that cysteamine inhibited the mutagenesis of nitrosodimethylamine (a highly toxic compound and known carcinogen in lab animals) in *Salmonella* TA100 and *E. coli* [77]. Goncharova and Kuzhir demonstrated the antimutagenic activity of cysteamine in Drosophila melanogaster against point mutations and chromosomal breakages induced by ethyl methanesulfonate, a direct-acting mutagen that induces mostly point mutations via alkylation of DNA [78]. These researchers found that cysteamine could suppress such mutagenicity at concentrations exceeding 0.125–0.250 M [77, 78]. In 1994, Watanabe et al. investigated the antimutagenic activity of cysteamine against 3-chloro-4-(dichloromethyl)-5-hydroxy-2(5H)-furanone (MX) in *Escherichia coli* cells [79]. MX is a direct-acting mutagen formed in chlorinated drinking water that has strong mutagenic activity in bacteria, accounting for about one-third to half of the total mutagenicity induced in bacteria by chlorinated tap water [80, 81]. Although MX has been shown to induce chromosomal aberrations in *in vitro* studies conducted on Chinese hamster ovary cells [82] and on rat peripheral lymphocytes [83], the compound has shown weak or no mutagenicity in *in vivo* studies conducted on mammalian cells. In this study, the researchers demonstrated effective inhibition of MX using cysteamine, suggesting that direct chemical inactivation of the mutagen is the responsible mechanism. Finally, Hoffman et al. investigated in 1995 the antimutagenic activity of cysteamine against bleomycin-induced mutations in yeast [84]. Bleomycin is a mutagenic chemotherapy agent known for inducing DNA damage in a variety of organisms, including micronuclei and chromosome aberrations in human lymphocytes [85, 86]. The researchers found that at concentrations equal to or above 16 mM and under hypoxic conditions, cysteamine significantly protected *Saccharomyces cerevisiae* from mitotic recombination induced by 6.25 *μ*g/ml bleomycin.

## 4. *In Vitro* Impacts on Tumoral Cells

Due to a lack of comprehensive evidence, the *in vitro* application of CysA in the treatment of tumors could not be categorized effectively.

Neural neoplastic cell lines are of great importance due to the inability of most chemotherapeutic agents to pass the blood–brain barrier. Given that CysA is a simple aminothiol that is readily distributed to the central nervous system, its efficacy has been tested on multiple neural neoplastic cell lines [87].

In 1996, the *in vitro* administration of CysA (for 72 hours) elicited the proliferation arrest of 2607 glioma cells (a neural neoplastic cell). CysA exposure time (at a lower IC_50_ of cysteamine) was due to duplication and lack of clarity. Magnifying the cell cycle, CysA slowed down the passage of 2607 cells through the S phase, leading to a cell cycle period prolongation followed by cell arrest at the G2/M phase. Normally, cell-cycling takes 24 h for 2607 cells. However, CysA-treated 2607 cells were kept in the cell cycle after 72 h, as indicated by a significant increase in 2607 cell density after 72 h of CysA exposure. In the same study, the cytostatic effect of CysA was observed *in vitro* on neuroblastoma cell lines (data has not been shown) [88].

Since CysA is a drug that passes the blood–brain barrier [66]; Jeitner et al. recommended that this drug could be used as a promising antitumor thiol for *in vivo* utilization in neural neoplastic disorders [88].

In a separate study, Jeitner et al. showed that CysA arrests CCRF-CEM and methotrexate-resistant leukemic cells. Cysteamine slowed down the passage of leukemic cells through the S phase, leading to proliferation arrest. Furthermore, cell viability decreased significantly (from ∼6.40 × 10^5^/mL to 3 × 10^5^/mL) after 24 h of treatment with CysA. Jeitner et al. recommended Cys as an antiproliferative agent for treatment of both drug naïve and drug resistant leukemia cells [87].

The physiochemical process behind both studies conducted by Jeitner et al. is suggested to have been H_2_O_2_ accumulation in cancer cell lines due to the metal chelating feature of CysA. The accumulation of H_2_O_2_ leads to the production of reactive radicals, which affects cell mitogenesis and proliferation. One other possible explanation of proliferation arrest is hypothesized as the ability of CysA to lessen DNA replication and synthesis. This mechanism was proven through measurements of the activity of two enzymes in DNA kinetics, namely, thymidine kinase and DNA polymerase [87, 88].

Since NO-releasing compounds have proven to be prominent cytotoxic and antitumor agents despite their unclear mechanisms of action [89], Zhukova et al. introduced a new NO-releasing tetranitrosyl binuclear [Fe-S] complex with CysA to induce human cancer cell apoptosis *in vitro*. NO exposure causes reactive NO production (RNOS); RNOS elicits DNA breaks via deamination and inactivation of human O^6^-methylguanine DNA methyltransferase (a key enzyme for genome stability). Meanwhile, CysA triggers cell apoptosis through activation of caspases 3 and 7. Taken together, NO-releasing compounds combined with CysA could be considered as crucial antitumor agents once preclinical trials are conducted [90].

## 5. Copper-Cysteamine: A New Sensitizer for Dynamic Cancer Therapies Induced by Light, X-Rays, Microwave, and Ultrasound Irradiations

Since the use of photosensitizer was shown to improve the efficiency of radiotherapy by reducing the amount of radiation needed to damage cancerous cells and thus reduce damages to adjacent tissues, CysA was used as component for a new generation of photosensitizers. Indeed, copper-cysteamine (Cu-CysA) nanoparticle is a novel photosensitizer that is considered to be promising.

The main assets of Cu-CysA nanoparticles are their ability to selectively target cancerous cells over normal cells as well as their low toxicity which means that Cu-CysA phototherapy is likely to lead to fewer side effects.

**Figure 3:**
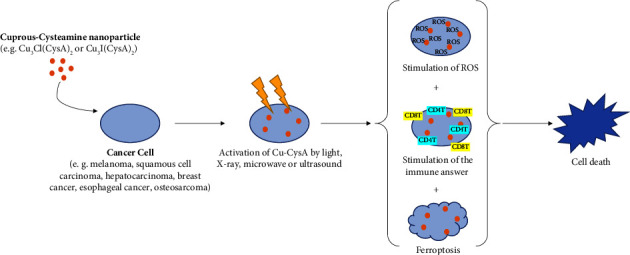
Schematic illustration of cuprous-cysteamine nanoparticle dynamic therapies induced by light, x-ray, microwave, or ultrasound irradiations.

Cu-CysA nanoparticles were shown to increase the levels of toxic singlet oxygens in cancerous cells during phototherapy (Figure 3). The singlet oxygens created by the activation of Cu-CysA using phototherapy are produced through what is considered to be a Fenton-like reaction. This reaction is favored by elevated levels of H_2_O_2_ and an acidic environment which are characteristic of tumor cells. Thus, using Cu-CysA, it is possible to selectively produce more singlet oxygens in cancer cells than in normal cells and therefore selectively killing cancer cells [91, 92]. Additionally, due to the enhanced permeability and retention (EPR) effect, Cu-CysA nanoparticles tend to accumulate more in tumor cells than in normal cells [92]. Thus, compared to other photosensitizers which tend to show nonselectivity and biological toxicity issues, Cu-CysA was chosen for both its selectivity and its low toxicity.

In addition to the production of singlet oxygens causing oxidative damage and leading to the direct destruction of the cancerous cell, Zhang et al. showed thanks to a melanoma model that the use of a Cu-CysA and X-ray combination could also inhibit tumor by inducing a strong antitumor immune response. This mechanism has shown to work through dendritic cells (DC) maturation which subsequently leads to the activation of CD4^+^T cells and CD8^+^T cells, as well as natural killer (NK) cells and the inhibition of M2 macrophages in the tumor's environment [93].

In a study from Liu et al. [94], the main mechanism of cell death observed in SW620 colorectal cells when using an X-ray activated Cu-CysA nanoparticles treatment was apoptosis; however, it also appeared that X-ray activated Cu-CysA nanoparticles induced autophagy.

The use of Cu-CysA nanoparticles conjugated with a pH-low insertion peptide (pHLIP) to treat cancer in a mice matrix was shown to improve the efficiency of the treatment and reduce tumor size compared to the use of plain Cu-CysA nanoparticles. It is theorized by Shrestha et al. that pHLIP strengthened the treatment by binding the nanoparticles directly to the cell and thus improving the efficiency of the singlet oxygens despite their short lifetimes [95].

Activation of Cu-CysA by microwaves has been evaluated by Yao and al. in 2016 and Hui et al. in 2023 [96, 97]. Microwave-induced photodynamic therapy presents the advantage to treat deeper-located cancer, such as bone cancers, as microwaves can penetrate deeper into tissues than most light, including near infrared [97]. After having showed that a concentration of 25 *μ*g/mL of Cu-CysA in combination with 10 min of MW irradiation at 20 W killed almost 100% of the osteosarcoma cancer cells, Yao et al. demonstrated in an *in vivo* model that the tumor growth significantly reduced with intratumoral injection of Cu-CysA at concentrations of 50 *μ*g/mL or 100 *μ*g/mL prior to microwave irradiation at 20W for 5 min [96]. Indication of tumor necrosis and suppression of cancer cell proliferation were observed on the tumor cells damaged by Cu-Cys and microwave irradiation [96]. As for X-ray activated Cu-CysA phototherapy, the production of singlet oxygen by activation under microwave radiation is involved in the cancer destruction, but it has not been clarified yet if those singlet oxygen were generated by microwave-induced heat or by the release of copper ions which in turn produced singlet oxygen [96].

Similar results were recently obtained on colorectal cancer cells: (i) all colorectal cancer cells were killed by combination of Cu-CysA at a concentration of 20 *μ*g/mL and 3 min of microwave irradiation at 20W; (ii) in a *in vivo* mouse model, the volume and size of tumor significantly reduced in the Cu-CysA + MW group compared to the control group [97]. However, in addition to the inhibition of the cell growth and the cell proliferation combined with cell death such as apoptosis and autophagy, another mechanism of action for microwave dynamic therapy was suggested; ferroptosis is a process in which ferrous ions oxidize polyunsaturated fatty acids by the action of ROS leading to lipid peroxidation. Additional studies are needed to fully understand the effect of ferroptosis-induced lipid peroxidation on intracellular membranes organelles and plasma membranes, but it is commonly accepted that ferroptosis leads to a nonapoptotic cell death [97, 98]. In the study from Zhou et al., several markers of ferroptosis, such as depletion of glutathione peroxide 4 (GPX4) and increase of lipid peroxides (LPO) and malondialdehyde (MDA), have been observed in cells treated with MWDT, demonstrating that MW-activated Cu-CysA killed colorectal cancer cells by ferroptosis [97].

The use of Cu-CysA nanoparticles in conjunction with ultrasound has also been evaluated and was shown to be effective to decrease cell viability of breast cancer cells in *in vitro* model [99]. In addition, repeated injection of Cu-CysA at concentration of 0.75 mg/kg prior ultrasound irradiation, in 4T1 tumor-bearing mice, showed a significant tumor growth reduction (74.43% of inhibition rate) compared to treatments with ultrasound alone (36.87%) or Cu-CysA alone (26.13%). A possible mechanism of Cu-CysA sonodynamic cancer treatment has been suggested; activation of Cu-CysA under ultrasound stimulated the generation of ROS leading to irreversible damage in tumor cells, such as DNA breaking, enhanced mitochondrial membrane permeability, and apoptosis [99].

Another compound used was a copper-cysteamine nanoparticles and disulfiram (DSF) combination. Similar to the other compound, Cu-CysA NPs produced toxic singlet oxygens through a Fenton-like reaction; however, due to the scavenging role of DSF, adding DSF leads to lower singlet oxygen concentrations than the use of plain Cu-CysA NPs. Despite this observation, the anticancer effects of the Cu-CysA + DSF combination were stronger than those of Cu-CysA NPs, likely because this new combination not only induced cancer cell directly through the production of singlet oxygen but also induced delayed cell death due to the toxicity of the DSF-Cu complex formed [100].

Most of the abovementioned studies [91, 92, 96, 97, 99, 100] were performed with Cu-CysA in the form of a crystal complex of formula Cu_3_Cl(SR)_2_ where R represents (CH_2_CH_2_NH_2_) [101]. Recent study from Wang et al. revealed that the exchange of the Cl-halogen by I- halogen forms a crystal which is more stable and has lower dark toxicity and improved performance on ROS generation under light and microwave irradiation [102]. This new Cu-CysA-Icuprous-cysteamine material opens the door for potential improved photodynamic therapies and chemotherapy.

Thus, CysA shows promising results as part of photosensitizers for radiotherapy.

## 6. Melanoma

Melanoma is a cancer that responds stubbornly to chemotherapy, mostly owing to its rapid tumoral cell proliferation and metastasis [103]. In dozens of studies, it has been established that the isolated or combined use of CysA or its derivatives can achieve a notable chemotherapeutic effect in the treatment of melanoma.

Catechol compounds (e.g., dopamine and 6-hydroxydopamine) are known antitumor agents for mouse and human subjects with melanoma. It is postulated that DNA polymerase inhibition in the melanoma tumor environment is the key mechanism of action of these compounds [104, 105]. Hydroquinone is another melanocytotoxic agent that has been used in the treatment of mouse melanoma. In fact, it has been demonstrated that hydroquinone increases the survival rate of melanoma-implanted mice. One possible explanation for this phenomenon is that hydroquinone induces melanocyte necrosis in the exposed tissue [106]. In a 1987 study, the chemotherapeutic efficacy of various catecholic and phenolic agents in combination with CysA or cysteine was tested on mice bearing melanoma. Amongst ten different agents, 4-S-cysteaminylphenol was shown to be a superior antimelanoma and melanocytotoxic agent. 4-S-cysteaminylphenol significantly prolonged the median survival time and life span of melanoma bearing mice. A combination of hydroquinone and cysteine (2-S-cysteinylhydroquinone) did not entail any longevity in mice life. 4-S-cysteaminylphenol appeared to significantly decrease the B16 melanoma tumor weight (64%) and volume in mice. As the second most effective antitumor compound in that study, 4-S-cysteinylphenol methyl ester exhibited inhibited the tumor growth (in terms of weight) by only 35.5%. In the same study, subcutaneous injection of 4-S-cysteaminylphenol in mice caused significant depigmentation of black hair. This effect lasted in the exposed area until the regrowth of white hair follicles. The mechanism of such action was demonstrated to be the malfunctioning of melanocytes (transferring melanosomes into keratinocytes) in the presence of 4-S-cysteaminylphenol. This gives an insight into a selective, melanocytotoxic effect of this agent. Overall, the mentioned study suggested 4-S-cysteaminylphenol as a more potent and less cytotoxic agent in the treatment of malignant melanoma compared with other chemotherapeutic agents [107].

The production of active oxygen radicals and systemic toxicity induced by catecholic compounds decrease their effectiveness as chemotherapeutic agents. Thus, phenolic compounds (precursors of catecholic compounds) seem to be more rational synthetic chemicals for use in the treatment of malignant melanoma.

4-S-Cysteaminylphenol (a melanin precursor) has been shown to have a potent *in vitro* killing effect on B16 melanoma cell lines. Markedly high melanin synthesis in malignant melanoma cell lines is known to be because of elevated tyrosinase activity (a rate-limiting enzyme in the melanin production cycle) [108]. This along with the fact that 4-S-cysteaminylphenol has zero efficacy in killing amelanotic melanoma cell lines suggest that the antimelanoma effect of 4-S-cysteaminylphenol is coordinated with oxidation by tyrosinase. It is also postulated that 4-S-cysteaminylphenol inhibits DNA synthesis through the inhibition of thymidine in melanin producing cells, which is another possible mechanism of action of this antimelanoma agent [109]. These data are in support of 4-S-cysteaminylphenol as an effective and rational antimelanoma agent.

In a 2010 study, CysA, as a simple and water-soluble aminothiol, was demonstrated to sensitize and kill B16 melanoma, doxorubicin-resistant MCF-7, and Hela cells in combination with doxorubicin (Dox). CysA + Dox resulted in 44.23% cancer cell death (eightfold increase over that of Dox alone). This means that CysA hastens the chemotherapeutic effect of Dox.

To assess the chemosensitization of CysA *in vivo*, Wan et al. examined 20 mice after implanting B16 melanoma cells subcutaneously and allowing the tumors to grow in size to about 50 mm^3^. Solo administration of Dox induced prominent tumor shrinkage, while CysA-Dox drastically decreased the tumor size, suggesting CysA to be a synergistic chemotherapy agent in combination with Dox [110].

It is documented that CysA chemosensitization is due to the formation of autophagosomes in exposed cells, which leads to a prodeath state. Increased autophagy seen in tumor cells that had undergone chemotherapy suggests this as an important mechanism of tumor cell killing [111]. Cysteamine has been shown to activate peroxidase-positive autophagosomes (Gomori bodies) in cultured human astrocytes through imposing structural changes in mitochondria, thereby inducing cell death [112].

Other combinatory uses of CysA derivatives have been covered in a wide range of antitumor studies both *in vivo* and *in vitro*. Perrimustine (CMSOEN2) is a CysA derivative proven to have a significant chemotherapeutic effect on B16 melanoma and metastasized melanoma cells by imposing DNA damage [113].

Based on these findings, CysA could be used to elicit an optimum chemotherapeutic effect in association with other agents in the treatment of melanoma. The lack of studies regarding the solitary use of CysA in the *in vivo* treatment of melanoma is worth mentioning.

## 7. Conclusion

Cysteamine (CysA) is ubiquitous in human body, able to pass the blood–brain barrier since it is a simple intermediate or byproduct of body metabolism that is readily distributed to all major organs including the human central nervous system. We have conducted a review of CysA's extensive effects on virtually all major organs and how they are put in medical use, either directly as a therapeutic agent for cystinosis and systemic lupus erythematosus (SLE) and neurodegenerative diseases or indirectly as an adjuvant in cancer therapies for an advanced disease, a regulator of mitotic cell growth and tumor activity, a potent antioxidant recommended for diseases in major organs linked with oxidative pathways, such as nonalcoholic fatty liver disease (NAFLD) and SLE, or as a radioprotective agent thanks to its antimutagenic properties against various types of radiation. In this review, the positive effects of CysA in the treatment of various cancer have been shown in *in vitro* and in *in vivo* studies: it prevented the apparition of new lesions in gastrointestinal, pancreatic, and hepatocellular cancer, decreased the number of colon tumors, limited the growth of sarcoma, and can be synergistically used in association with other agents in the treatment of melanoma. Overall, from the reports available, it can be concluded that the main mechanism by which cysteamine provides anticancer and radioprotection is the scavenging of free radicals. On the flip side of these, CysA in the form of Cu-CysA nanoparticle has been suggested as a novel sensitizer for dynamic cancer therapies induced by light, X-rays, microwave, and ultrasound irradiations; under irradiation, Cu-CysA can selectively target tumoral cells and stimulate inside them the generation of ROS leading to apoptic or nonapoptic cell death. The ability of cysteamine to respectively scavenge or stimulate the generation of free radicals, if respectively used alone or in the form of Cu-CysA and under irradiation, is somewhat unique and opens the way to various anticancer applications.

Although many *in vitro* and *in vivo* studies have recommended CysA- and Cu-CysA-based formulations as a prominent antitumor and antimutagenic agent for decades, the discussion over their exact mechanisms of action is still growing. It seems that too many facets of CysA and Cu-Cu-CysA remain masked. Further research is required to determine the precise mechanisms of cysteamine's antimutagenicity against various mutagens. Considering the long history of safety, the lack of *in vivo* human models and clinical trials to make the final decision of how CysA must be used is also worth mentioning. In conclusion, cysteamine is a promising, safe agent that acts via various pathways to either prevent or treat different cancers.

## Data Availability

No data were used in this study.

## References

[1] Atallah C., Charcosset C., Greige-Gerges H. (2020). Challenges for cysteamine stabilization, quantification, and biological effects improvement. *Journal of Pharmaceutical Analysis*.

[2] Paul B. D., Snyder S. H. (2019). Therapeutic applications of cysteamine and cystamine in neurodegenerative and neuropsychiatric diseases. *Frontiers in Neurology*.

[3] Besouw M., Masereeuw R., van den Heuvel L., Levtchenko E. (2013). Cysteamine: an old drug with new potential. *Drug Discovery Today*.

[4] Aruoma O. I., Halliwell B., Hoey B. M., Butler J. (1988). The antioxidant action of taurine, hypotaurine and their metabolic precursors. *Biochemical Journal*.

[5] Wood P. L., Khan M. A., Moskal J. R. (2007). Cellular thiol pools are responsible for sequestration of cytotoxic reactive aldehydes: central role of free cysteine and cysteamine. *Brain Research*.

[6] Kessler A., Biasibetti M., da Silva Melo D. A. (2008). Antioxidant effect of cysteamine in brain cortex of young rats. *Neurochemical Research*.

[7] Naquet P., Pitari G., Dupre S., Galland F. (2014). Role of the Vnn1 pantetheinase in tissue tolerance to stress. *Biochemical Society Transactions*.

[8] Okamura D. M., Himmelfarb J. (2009). Tipping the redox balance of oxidative stress in fibrogenic pathways in chronic kidney disease. *Pediatric Nephrology*.

[9] Okamura D. M., Bahrami N. M., Ren S. (2014). Cysteamine modulates oxidative stress and blocks myofibroblast activity in CKD. *Journal of the American Society of Nephrology*.

[10] Bai M., Liu H., Xu K. (2018). Effects of dietary coated cysteamine hydrochloride on pork color in finishing pigs. *Journal of the Science of Food and Agriculture*.

[11] Takahashi M., Shirai T., Fukushima S., Hahanouchi M., Hirose M., Ito N. (1976). Effect of fundic ulcers induced by iodoacetamide on development of gastric tumors in rats treated with N-methyl-N’-nitro-Nnitrosoguanidine. *Gann*.

[12] Takahashi M., Shirai T., Fukushima S. (1981). Ulcer formation and associated tumor production in multiple sites within the stomach and duodenum of rats treated with N-methyl-N’-nitro-N-nitrosoguanidine. *Journal of the National Cancer Institute*.

[13] Shirai T., Takahashi M., Fukushima S., Tatematsu M., Hirose M., Ito N. (1978). Induction of preneoplastic hyperplasia and carcinoma by N-methyl-N’-nitro-N-nitroso guanidine from regenerated mucosa of ulcers induced by iodoacetamide in fundus of rat stomach. *Gann*.

[14] Watanabe H. (2010). Intestinal metaplasia-the effect of Acid on the gastric mucosa and gastric carcinogenesis. *Journal of Toxicologic Pathology*.

[15] Sasajima K., Kawachi T., Matsukura N., Sano T., Sugimura T. (1979). Intestinal metaplasia and adenocarcinoma induced in the stomach of rats by N-propyl-N′-nitro-N-nitrosoguanidine. *Journal of Cancer Research and Clinical Oncology*.

[16] Watanabe H., Kamikawa M., Nakagawa Y., Takahashi T., Ito A. (1988). The effects of ranitidine and cysteamine on intestinal metaplasia induced by X-irradiation in rats. *Pathology International*.

[17] Fujii I., Watanabe H., Naito M., Kawashima K., Ito A. (1985). The induction of intestinal metaplasia in rats by pyloroplasty or pyloroplasty plus vagotomy. *Pathology, Research and Practice*.

[18] Lichtenberger L. M., Szabo S., Trier J. S. (1977). Duodenal ulcerogens, cysteamine and propionitrile, stimulate serum gastrin levels in the rat. *Gastroenterology*.

[19] Ishii Y., Fujii Y., Homma M. (1976). Gastric acid stimulating action of cysteamine in the rat. *European Journal of Pharmacology*.

[20] Kirkegaard P., Poulsen S. S., Loud F. B., Halse C., Christiansen J. (1980). Cysteamine-induced duodenal ulcer and acid secretion in the rat. *Scandinavian Journal of Gastroenterology*.

[21] Tatsuta M., Iishi H., Yamamura H., Baba M., Mikuni T., Taniguchi H. (1988). Inhibitory effect of prolonged administration of cysteamine on experimental carcinogenesis in rat stomach induced by N-methyl-N′-nitro-N-nitrosoguanidine. *International Journal of Cancer*.

[22] Tatsuta M., Iishi H., Baba M., Mikuni T., Taniguchi H. (1989). Effects of propranolol and cimetidine on cysteamine inhibition of gastric carcinogenesis induced in wistar rats by n-methyl-n′-nitro-n-nitrosoguanidine. *International Journal of Cancer*.

[23] Canfield S. P., Hughes A. D., Price C. A., Spencer J. E. (1981). The action of beta-adrenoceptor agonists on acid secretion by the rat isolated stomach. *Journal of Physiology*.

[24] Henriksson R., Zachrisson B., Blom H., Hellström S. (1984). Gastric parietal cell changes due to long-term propranolol administration in adult and developing rats. *The Lancet*.

[25] Mallory T. B. (1940). Carcinoma in situ of the stomach and its bearing on the histogenesis of malignant ulcers. *Archives of Pathology and Laboratory Medicine*.

[26] Palmer W. L. (1944). Gastric carcinoma. observations on peptic ulceration & healing. *Gastroenterology*.

[27] Koh M., Takahashi T., Kurokawa Y. (2021). Propranolol suppresses gastric cancer cell growth by regulating proliferation and apoptosis. *Gastric Cancer*.

[28] Gallagher G. T., Szabo S. (1984). Secretory changes associated with chemically-induced duodenal ulceration: simultaneous measurements of acid, pepsin, base and pancreatic enzymes in rats with chronic gastric fistula. *Digestion*.

[29] Johnson L. R. (1977). New aspects of the trophic action of gastrointestinal hormones. *Gastroenterology*.

[30] Badger T. M., Sagar S. M., Millard W. J., Martin J. B., Rosenblum P. (1982). Cysteamine reduces serum gonadotropin concentrations in adult male rats. *Life Sciences*.

[31] Szabo S., Reichlin S. (1983). Somatostatin depletion of the gut and pancreas induced by cysteamine is not prevented by vagotomy or by dopamine agonists. *Regulatory Peptides*.

[32] Tatsuta M., Itoh T., Okuda S. (1980). Effects of gastrin and histamine on gastric carcinogenesis induced in rats by N-methyl-N′-nitro-N-nitrosoguanidine. *European Journal of Cancer*.

[33] Näslund M., Ehrenberg L., Djalali-Behzad G. (1976). Antagonism of ascorbate against the radioprotective action of cysteamine. *International Journal of Radiation Biology & Related Studies in Physics, Chemistry & Medicine*.

[34] Tatsuta M., Iishi H., Baba M., Taniguchi H. (1989). Tissue norepinephrine depletion as a mechanism for cysteamine inhibition of colon carcinogenesis induced by azoxymethane in Wistar rats. *International Journal of Cancer*.

[35] Tutton P. J. M., Barkla D. H. (1977). The influence of adrenoceptor activity on cell proliferation in colonic crypt epithelium and in colonic adenocarcinomata. *Virchows Archiv-B: Cell Pathology*.

[36] Tutton P. J. M., Barkla D. H. (1976). A comparison of cell proliferation in normal and neoplastic intestinal epithelia following either biogenic amine depletion or monoamine oxidase inhibition. *Virchows Archiv-B: Cell Pathology*.

[37] Szabo S., Horner H. C., Maull H., Schnoor J., Chiueh C. C., Palkovits M. (1987). Biochemical changes in tissue catecholamines and serotonin in duodenal ulceration caused by cysteamine or propionitrile in the rat. *Journal of Pharmacology and Experimental Therapeutics*.

[38] Takagi Y., Shikita M. (1983). Cysteamine rapidly decreases mitotic cells in random culture of HeLa S-3 cells. *Journal of Radiation Research*.

[39] Ilic M., Ilic I. (2016). Epidemiology of pancreatic cancer. *World Journal of Gastroenterology*.

[40] Fujisawa T., Rubin B., Suzuki A. (2012). Cysteamine suppresses invasion, metastasis and prolongs survival by inhibiting matrix metalloproteinases in a mouse model of human pancreatic cancer. *PLoS One*.

[41] Chang C., Werb Z. (2001). The many faces of metalloproteases: cell growth, invasion, angiogenesis and metastasis. *Trends in Cell Biology*.

[42] Basset P., Okada A., Chenard M. P. (1997). Matrix metalloproteinases as stromal effectors of human carcinoma progression: therapeutic implications. *Matrix Biology*.

[43] Kilian M., Gregor J. I., Heukamp I. (2006). Matrix metalloproteinase inhibitor RO 28-2653 decreases liver metastasis by reduction of MMP-2 and MMP-9 concentration in BOP-induced ductal pancreatic cancer in Syrian Hamsters: inhibition of matrix metalloproteinases in pancreatic cancer. *Prostaglandins, Leukotrienes and Essential Fatty Acids*.

[44] Suzuki A., Bhardwaj R., Leland P., Joshi B. H., Puri R. K. (2017). Abstract 4900: cysteamine suppresses tumor metastasis by inhibiting activity of matrix metalloproteases without inducing toxicity in mouse models of human ovarian cancer. *Cancer Research*.

[45] Tatsuta M., Iishi H., Baba M. (1989). Inhibition by cysteamine of hepatocarcinogenesis induced by N-nitrosomorpholine in sprague-dawley rats. *International Journal of Cancer*.

[46] Millard W. J., Sagar S. M., Badger T. M., Martin J. B. (1983). Cysteamine effects on growth hormone secretion in the male rat. *Endocrinology*.

[47] Parsons J. A., Peterson E. K., Hartfel M. A. (1984). Effects of cysteamine on pituitary, MtTW15 tumor, and serum prolactin levels measured by rat lymphoma cell bioassay and radioimmunoassay. *Endocrinology*.

[48] Cruise J. L., Knechtle S. J., Bollinger R. R., Kuhn C., Michalopoulos G. (1987). *α*1-Adrenergic effects and liver regeneration. *Hepatology*.

[49] Cruise J. L., Houck K. A., Michalopoulos G. (1988). Early events in the regulation of hepatocyte DNA synthesis: the role of alpha-adrenergic stimulation. *Scandinavian Journal of Gastroenterology*.

[50] Gomaa A. I., Khan S. A., Toledano M. B., Waked I., Taylor-Robinson S. D. (2008). Hepatocellular carcinoma: epidemiology, risk factors and pathogenesis. *World Journal of Gastroenterology*.

[51] Dohil R., Meyer L., Schmeltzer S., Cabrera B. L., Lavine J. E., Phillips S. A. (2012). The effect of cysteamine bitartrate on adiponectin multimerization in non-alcoholic fatty liver disease and healthy subjects. *The Journal of Pediatrics*.

[52] Dohil R., Schmeltzer S., Cabrera B. L. (2011). Enteric-coated cysteamine for the treatment of paediatric non-alcoholic fatty liver disease. *Alimentary Pharmacology and Therapeutics*.

[53] Hsu T. C., Huang C. Y., Chiang S. Y., Lai W. X., Tsai C. H., Tzang B. S. (2008). Transglutaminase inhibitor cystamine alleviates the abnormality in liver from NZB/W F1 mice. *European Journal of Pharmacology*.

[54] Ghoncheh M., Pournamdar Z., Salehiniya H. (2016). Incidence and mortality and epidemiology of breast cancer in the world. *Asian Pacific Journal of Cancer Prevention*.

[55] Inano H., Onoda M. (2002). Prevention of radiation-induced mammary tumors. *International Journal of Radiation Oncology, Biology, Physics*.

[56] Henderson B. W., Miller A. C. (1986). Effects of scavengers of reactive oxygen and radical species on cell survival following photodynamic treatment in vitro: comparison to ionizing radiation. *Radiation Research*.

[57] Marquardt H., Sapozink M. D., Zedeck M. S. (1974). Inhibition by cysteamine-HCl of oncogenesis induced by 7,12-dimethylbenz(alpha)anthracene without affecting toxicity. *Cancer Research*.

[58] Inano H., Onoda M., Suzuki K., Kobayashi H., Wakabayashi K. (2000). Inhibitory effects of WR-2721 and cysteamine on tumor initiation in mammary glands of pregnant rats by radiation. *Radiation Research*.

[59] Scott J. S., Lakin C. A., Oliver J. R. (1987). The effect of cysteamine, cystamine, and the structurally related compounds taurine, N-acetyl-cysteine, and D-penicillamine on plasma prolactin levels in normal and estrogen-primed hyperprolactinemic rats. *Endocrinology*.

[60] Suzuki K., Ishi-Ohba H., Yamanouchi H., Wakabayashi K., Takahashi M., Inano H. (1994). Susceptibility of lactating rat mammary glands to gamma-ray-irradiation-induced tumorigenesis. *International Journal of Cancer*.

[61] Inano H., Yamanouchi H., Suzuki K., Onoda M., Wakabayashi K. (1995). Estradiol-17*β* as an initiation modifier for radiation-induced mammary tumorigenesis of rats ovariectomized before puberty. *Carcinogenesis*.

[62] Inano H., Suzuki K., Ishii-Ohba H., Ikeda K., Wakabayashi K. (1991). Pregnancy-dependent initiation in tumorigenesis of Wistar rat mammary glands by 60Co-irradiation. *Carcinogenesis*.

[63] Gage M. M., Nagarajan N., Ruck J. M. (2019). Sarcomas in the United States: recent trends and a call for improved staging. *Oncotarget*.

[64] Casazza A., Di Conza G., Wenes M., Finisguerra V., Deschoemaeker S., Mazzone M. (2014). Tumor stroma: a complexity dictated by the hypoxic tumor microenvironment. *Oncogene*.

[65] Giessner C., Millet V., Mostert K. J. (2018). Vnn1 pantetheinase limits the Warburg effect and sarcoma growth by rescuing mitochondrial activity. *Life Science Alliance*.

[66] Devik F., Lothe F. (1955). The effect of cysteamine, cystamine and hypoxia on mortality and bone marrow chromosome aberrations in mice after total body roentgen irradiation. *Acta Radiologica*.

[67] Lüning K. G., Frölén H., Nelson A., Luning K. G., Frolen H. (1961). The protective effect of cysteamine against genetic damages by X-rays in spermatozoa from mice. *Radiation Research*.

[68] Kølmark H. G. (1965). Protection by 2-mercaptoethylamine against the mutagenic and lethal effects of X-rays in Neurospora crassa. *Mutation Research: Fundamental and Molecular Mechanisms of Mutagenesis*.

[69] Vos O., Kaalen M. C. (1968). Protection against ionizing radiation at the cellular level, assessed by various parameters. *International Journal of Radiation Biology and Related Studies in Physics, Chemistry & Medicine*.

[70] Mikaelsen K., Pedersen K. (2009). Protective effectiveness of cysteine, cysteamine and cystamine against x-ray induced chromosome aberrations at different stages of mitosis. *Hereditas*.

[71] Lohman P. H. M., Vos O., Van Sluis C. A., Cohen J. A. (1970). Chemical protection against breaks induced in DNA of human and bacterial cells by X-irradiation. *Biochimica et Biophysica Acta (BBA)-Nucleic Acids and Protein Synthesis*.

[72] Roots R., Okada S. (1972). Protection of DNA molecules of cultured mammalian cells from radiation-induced single-strand scissions by various alcohols and SH compounds. *International Journal of Radiation Biology and Related Studies in Physics, Chemistry and Medicine*.

[73] Stern M. G., Van Dillewijn J., Blok J. (1968). The influence of some sulphydryl compounds on *γ*-ray-induced inactivation and reversion to prototrophy in *E. coli* B fil-citrul. *Mutation Research: Fundamental and Molecular Mechanisms of Mutagenesis*.

[74] Radman M., Roller A., Errera M. (1969). Protection and host cell repair of irradiated lambda phage. *Molecular and General Genetics MGG*.

[75] Becher R., Gebhart E. (1973). Die Schutzwirkung von Cysteamin und fl-Aminoi∼thylisothiouronium (AET) gegen die chromosomenseh∼digende Aktivit∼t yon Trenimon ® in mensehlichen Lymphocyten in vitro. *Humangenetik*.

[76] Rosin M. P., Stich H. F. (1978). Inhibitory effect of reducing agents on N-acetoxy-and N-hydroxy-2-acetylaminofluorene-induced mutagenesis. *Cancer Research*.

[77] Negishi T., Ohara Y., Hayatsu H. (1982). *A Sensitive Assay for Mutagenic Activity of N-Nitrosamines and its Use for Detection of Modulators of the Mutagenicity*.

[78] Goncharova R. I., Kuzhir T. D. (1989). A comparative study of the antimitagenic effects of antioxidants on chemical mutagenesis in *Drosophila melanogaster*. *Mutation Research: Fundamental and Molecular Mechanisms of Mutagenesis*.

[79] Watanabe M., Kobayashi H., Ohta T. (1994). Rapid inactivation of 3-chloro-4-(dichloromethyl)-5-hydroxy-2 (5H)-furanone (MX), a potent mutagen in chlorinated drinking water, by sulfhydryl compounds. *Mutation Research: Environmental Mutagenesis & Related Subjects*.

[80] Kronberg L., Vartiaine T. (1988). Ames mutagenicity and concentration of the strong mutagen 3-chloro-4-(dichloromethyl)-5-hydroxy-2(5H)-furanone and of its geometric isomer E-2-chloro-3-(dichloromethyl)-4-oxobutenoic acid in chlorine-treated tap waters. *Mutation Research: Genetic Toxicology*.

[81] Kronberg L., Holmbom B., Reunanen M., Tikkanen L. (1988). Identification and quantification of the Ames mutagenic compound 3-chloro-4-(dichloromethyl)-5-hydroxy- 2(5H)-furanone and of its geometric isomer (E)-2-chloro-3-(dichloromethyl)-4-oxobutenoic acid in chlorine-treated humic water and drinking water extracts. *Environmental Science and Technology*.

[82] Meier J. R., Blazak W. F., Knohl R. B. (1987). Mutagenic and clastogenic properties of 3-chloro-4-(dichloromethyl)-5-hydroxy-2 (5H)-furanone: a potent bacterial mutagen in drinking water. *Environmental and Molecular Mutagenesis*.

[83] Jansson K., Mäki-Paakkanen J., Vaittinen S. L., Vartiainen T., Komulainen H., Tuomisto J. (1993). Cytogenetic effects of 3-chloro-4-(dichloromethyl)-5-hydroxy-2 (50H)-furanone (MX) in rat peripheral lymphocytes in vitro and in vivo. *Mutation Research: Genetic Toxicology*.

[84] Hoffmann G. R., Quaranta J. L., Shorter R. A., Littlefield L. G. (1995). Modulation of bleomycin-induced mitotic recombination in yeast by the aminothiols cysteamine and WR-1065. *Molecular and General Genetics MGG*.

[85] Dresp J., Schmid E., Bauchinger M. (1978). The cytogenetic effect of bleomycin on human peripheral lymphocytes in vitro and in vivo. *Mutation Research: Fundamental and Molecular Mechanisms of Mutagenesis*.

[86] Hoffmann G. R., Colyer S. P., Littlefield L. G. (1993). Induction of micronuclei by bleomycin in G0 human lymphocytes: I. Dose-response and distribution. *Environmental and Molecular Mutagenesis*.

[87] Jeitner T. M., Renton F. J. (1996). Inhibition of the proliferation of human neural neoplastic cell lines by cysteamine. *Cancer Letters*.

[88] Jeitner T. M., Delikatny E. J., Bartier W. A., Capper H. R., Hunt N. H. (1998). Inhibition of drug-naive and-resistant leukemia cell proliferation by low molecular weight thiols. *Biochemical Pharmacology*.

[89] Kielbik M., Klink M., Brzezinska M., Szulc I., Sulowska Z. (2013). Nitric oxide donors: spermine/NO and diethylenetriamine/NO induce ovarian cancer cell death and affect STAT3 and AKT signaling proteins. *Nitric Oxide*.

[90] Zhukova O. S., Smirnova Z. S., Chikileva I. O., Kiselevskii M. V. (2017). Antiproliferative activity of a new nitrosyl iron complex with cysteamine in human tumor cells in vitro. *Bulletin of Experimental Biology and Medicine*.

[91] Chudal L., Pandey N. K., Phan J. (2020). Copper-cysteamine nanoparticles as a heterogeneous fenton-like catalyst for highly selective cancer treatment. *ACS Applied Bio Materials*.

[92] Chen X., Liu J., Li Y. (2022). Study of copper-cysteamine based X-ray induced photodynamic therapy and its effects on cancer cell proliferation and migration in a clinical mimic setting. *Bioactive Materials*.

[93] Zhang Q., Guo X., Cheng Y. (2020). Use of copper-cysteamine nanoparticles to simultaneously enable radiotherapy, oxidative therapy and immunotherapy for melanoma treatment. *Signal Transduction and Targeted Therapy*.

[94] Liu Z., Xiong L., Ouyang G., Ma L., Sahi S., Wang K. (2017). Investigation of copper cysteamine nanoparticles as a new type of radiosensitisers for colorectal carcinoma treatment. *Scientific Reports*.

[95] Shrestha S., Wu J., Sah B. (2019). X-ray induced photodynamic therapy with copper-cysteamine nanoparticles in mice tumors. *Proceedings of the National Academy of Sciences*.

[96] Yao M., Ma L., Li L. (2016). A new modality for cancer treatment—nanoparticle mediated microwave induced photodynamic therapy. *Journal of Biomedical Nanotechnology*.

[97] Zhou H., Liu Z., Zhang Z. (2023). Copper-cysteamine nanoparticle-mediated microwave dynamic therapy improves cancer treatment with induction of ferroptosis. *Bioactive Materials*.

[98] Chen X., Li J., Kang R., Klionsky D. J., Tang D. (2021). Ferroptosis: machinery and regulation. *Autophagy*.

[99] Wang P., Wang X., Ma L. (2018). Nanosonosensitization by using copper–cysteamine nanoparticles augmented sonodynamic cancer treatment. *Particle a Particle Systems Characterization*.

[100] Chang Y., Wu F., Pandey N. K. (2020). Combination of disulfiram and copper–cysteamine nanoparticles for an enhanced antitumor effect on esophageal cancer. *ACS Applied Bio Materials*.

[101] Ma L., Chen W., Schatte G. (2014). A new Cu–cysteamine complex: structure and optical properties. *Journal of Materials Chemistry C*.

[102] Wang Y., Alkhaldi N. D., Pandey N. K. (2021). A new type of cuprous-cysteamine sensitizers: synthesis, optical properties and potential applications. *Materials Today Physics*.

[103] Divito S. J., Ferris L. K. (2010). Advances and short comings in the early diagnosis of melanoma. *Melanoma Research*.

[104] Wick M. M. (1979). 3, 4-Dihydroxybenzylamine: a dopamine analog with enhanced antitumor activity against B16 melanoma. *Journal of the National Cancer Institute*.

[105] Wick M. M. (1980). Levodopa and dopamine analogs as DNA polymerase inhibitors and antitumor agents in human melanoma. *Cancer Research*.

[106] Jimbow K., Obata H., Pathak M. A., Fitzpatrick T. B. (1974). Mechanism of depigmentation by hydroquinone. *Journal of Investigative Dermatology*.

[107] Miura S., Ueda T., Jimbow K., Ito S., Fujita K. (1987). Synthesis of cysteinylphenol, cysteaminylphenol, and related compounds, and in vivo evaluation of antimelanoma effect. *Archives of Dermatological Research*.

[108] Pawelek J. M. (1976). Factors regulating growth and pigmentation of melanoma cells. *Journal of Investigative Dermatology*.

[109] Yamada I., Seki S., Ito S., Suzuki S., Matsubara O., Kasuga T. (1991). The killing effect of 4-S-cysteaminylphenol, a newly synthesised melanin precursor, on B16 melanoma cell lines. *British Journal of Cancer*.

[110] Wan X. M., Zheng F., Zhang L., Miao Y. Y., Man N., Wen L. P. (2011). Autophagy-mediated chemosensitization by cysteamine in cancer cells. *International Journal of Cancer*.

[111] Kondo Y., Kanzawa T., Sawaya R., Kondo S. (2005). The role of autophagy in cancer development and response to therapy. *Nature Reviews Cancer*.

[112] Brawer J. R., Reichard G., Small L., Schipper H. M. (1994). The origin and composition of peroxidase-positive granules in cysteamine-treated astrocytes in culture. *Brain Research*.

[113] Godeneche D., Rapp M., Thierry A. (1990). DNA damage induced by a new 2-chloroethyl nitrosourea on malignant melanoma cells. *Cancer Research*.

